# Comment on Postma et al. Predicted Public Health and Economic Impact of Respiratory Syncytial Virus Vaccination with Variable Duration of Protection for Adults ≥60 Years in Belgium. *Vaccines* 2023, *11*, 990

**DOI:** 10.3390/vaccines11111646

**Published:** 2023-10-27

**Authors:** Baudouin Standaert

**Affiliations:** 1 Department Care and Ethics, Faculty of Medicine and Life Sciences, University Hasselt, 3590 Diepenbeek, Belgium; baudouin.standaert@uhasselt.be; 2 HEBO bv, 2020 Antwerpen, Belgium

Presently, there are at least five important vaccine producers that have already launched or intend to launch a new vaccine designed to prevent infections caused by the Respiratory Syncytial Virus (RSV), which is highly prevalent in the youngest as well as the oldest age groups [1]. Among those vaccines is the vaccine of GSK RSVPreF3-OA (Arexvy), which is an adjuvanted vaccine with an indication for people of 60 years (yr) old (OA) or more [2]. The Pfizer vaccine is called Abrysvo and is a bivalent RSV-A and RSV-B prefusion F subunit vaccine with the indication to prevent RSV infections and hospitalizations in infants and older adults [3]. For infants, the Pfizer vaccine is designed to stimulate the production of serum anti-F immunoglobulin G in the mother, which can then be transferred to the fetus across the placenta and protected for the first six months of life when the risk of hospitalization is highest. For seniors (60 years+), the vaccination prevents acute respiratory disease and lower respiratory tract disease caused by RSV. Both vaccines, from GSK and Pfizer, have been approved by the FDA for use in 60 years+ and recommended by the ACIP recently (21 June 2023) [4]. The vaccine of GSK has also been endorsed by the European Medicines Agency (EMA) and was approved by the European Commission on 7 June 2023. Moderna and Bavarian Nordic are attempting to join the pool of available RSV vaccines in the coming months with slightly different compositions in their vaccine proposal, mRNA for Moderna and MVA (Modified Vaccinia Ankara virus) for Bavarian Nordic [1].

It is quite unique that so many manufacturers are marketing their product at approximately the same time. During the COVID-19 pandemic, different producers developed new vaccines around the same time, but that was a forced scenario in a setting of a completely novel pandemic infection with new prevalence and incidence data every day. Here, the RSV infection is an existing and well-known illness occurring in a regular annual pattern: a seasonal event which peaks during the winter period each year [5]. It is therefore most comparable to influenza, which is partially under control with large-scale vaccination programs [6]. 

Additionally, the pattern of RSV among infants and children compared to the oldest age groups is different because the latter are exposed to chronic illnesses, which may aggravate the infection [7]. This is the reason the disease burden is often assessed separately for each age group even though the spread of the virus can be influenced by transmission from young to old, as seen in other respiratory infections [8]. 

Getting a new vaccine approved for reimbursement in a country is a very urgent matter for any company who wish to be the first on the market since being the first helps define the price of the vaccine. Within that framework, a high disease burden without vaccination—linked with a large vaccine impact on the disease—augments the chances of obtaining a higher reimbursement price for the vaccine [9]. As it remains unknown at launch the short- to long-term effect of the vaccination program, predictive models operating under specific conditions (cohort or population models) are relied on to estimate the long duration effect of the vaccine. Consequently, there is pressure on the shoulders of epidemiologists and health economists in a vaccine company to produce credible results and to also publish these data to achieve greater acceptance by all stakeholders. Only then is it possible to know what an acceptable price for the vaccine could be. 

This is what the publication of the Johnson & Johnson (J&J) group tries to achieve in their latest assessment, as presented in this journal [10]. Other companies like Pfizer, Merck, Moderna, GSK, Novavax, or Sanofi—to name a few—have conducted a sort of analysis taken from different angles and using different regions, but unfortunately this does not facilitate a comparison of their findings [11,12,13,14,15,16]. As an example, Pfizer presented a systematic review of the literature about the epidemiologic prevalence of the disease worldwide for a period before RSV was more intensely researched for, published in 2020 [11], while Moderna conducted a systematic literature review on the costs of the disease only for a more recent period, with no reference to the epidemiologic data [12]. Given this reality, there are two questions one should ask when examining published information sponsored and developed by the vaccine industry at this stage, which is mainly searching for indications that the disease burden is underreported and huge while the client—the authorities—is most interested in a transparent and honest view of the disease burden compared with other frequently occurring respiratory infectious diseases. First, we ask what is the value of the information disclosed in the paper under discussion: are the data complete and accurate enough to effectively submit that information to the Belgian authorities for assigning the reimbursement price of their new vaccine? This question will be answered in greater detail in later paragraphs. 

The second question is to ask how far their evaluation deviates from the data published by other companies? The problem is that each company is conducting their own separate evaluations utilizing different data and methods, despite claiming that it is a systematic review, whereas under such circumstance, each analysis should ideally result in the generation of the same numbers. The burden of disease in a particular country should not be different depending on which company is conducting the analysis. If there are differences, it is then important to investigate where the differences lie, why, and by how much. 

Regarding the first question, the starting point for designing an exercise that defines the burden of disease on a national level should be framed in accordance with the guidelines which have been set up by the local authorities. For Belgium, those guidelines are well known and were published in 2012, providing a clear framework for what to disclose regarding the disease burden [17]. These guidelines help to indicate what could be absent in the publication, here discussed, that is needed to achieve a complete economic analysis of the potential impact of the new vaccine. 

More specifically, the health effect of the disease (as expressed in QALYs lost or DALYs obtained) is an essential part of this assessment, but unfortunately it was not mentioned in the paper, while some estimates on the QALY-loss have been reported recently [18]. Mainly costing data were presented, which would be suitable if the intention was a budget-only exercise. However, since the authors mentioned the terms “public health” and “economic impact” in their title, their work should have included a broad assessment about how the aging population normally behaves in relation to health and healthcare consumption, as expressed through the use of home care, primary health care, day care units, nursing homes, and hospital care. In this case, mainly hospital care and primary healthcare were the primary outcomes measured and reported. The term “economic impact” indicates that the authors were interested in searching not only for costing data but also for specific health assessments (direct and indirect); but, as mentioned earlier, this was not conducted. 

Another unmentioned element in the paper regarding the economic impact was that no cost for the RSV vaccine was included in the equation when the literature referenced in the paper could have supplied that information [19]. The authors therefore claimed direct medical cost “savings” in the vaccination scenario, but that is difficult to argue if the cost of the vaccine is not considered. 

There are a few other strange elements observed in the calculations made. The authors conducted a pooled analysis of the age group 60+ even though there is strong heterogeneity present, e.g., age, sex, co-morbidity spread, and location, factors which are all involved in varied exposures to the infection [20]. Their model structure (Figure 1) shows a population overall mortality impact separated from the vaccine and the no-vaccine branches, which is bizarre if the vaccine arm avoids more specific deaths than the no-vaccine arm. 

No adjustment was made for the important hospital cost difference in geriatric versus pulmonology wards in Belgium, where a geriatric patient receives a reimbursement of 13 days on average, while the pulmonology ward received only 6 days, and that there are more geriatric beds that can still augment in numbers year after year, while the amount of pulmonology beds are reduced every year [21]. Indirect costs were not separately assessed for hospitalized patients of 60 to 64 years old (still working), but they were assumed to be retired 65+ people. Lastly, a discount rate was applied to the cost when only a cost/budget evaluation was presented. 

In short, the impression given was that the intention of the paper was to produce a rapid first cost estimate about the disease burden of RSV among aging adults impacted by the vaccine mid-term. However, decision makers like to see greater granularity with more precise estimates related to the effects of a vaccine on the cost of illness and other health outcomes. These values are evaluable for RSV disease, but authorities also like to see the differences in a comparison with other respiratory infections for which vaccines are used (e.g., flu, pneumococcal infections, and COVID-19) in the same age group. This total package of information was not disclosed in the paper as it was not the initial focus. However, a good starting point for conducting the exercise should have been to consider what is it that authorities would like to see and to know about the disease, the vaccine, and ageing adults in an overall context of respiratory infectious diseases. It will, however, never be known whether the published data were actually sufficient for the Belgian authorities since J&J decided a few months ago to step down from the RSV vaccine race for the elderly [22].

This brings us back to the second question mentioned earlier about potential deviations in the reported data by each industrial group, which therefore raises the following issue: why is the burden of disease calculated by different groups in an isolated, stand-alone fashion instead of collaborating between them to obtain more precise information, crucial to further analyses? When all is said and done, the numbers regarding the burden of the disease, whether it is the epidemiology or the cost results, should be the same regardless of who is conducting the exercise because those data should not be variable depending on the vaccine used. It would be better if a uniform, homogeneous protocol for investigation and data collection would have been developed by all who are concerned with the prevention of RSV infection, which would likely increase the credibility of the work presented to local authorities. 

With that in mind, the European Commission, with its known Horizon 2020 research program, introduced in 2005 instruments to enhance close collaboration between the public and private sectors, like academia groups and industry. It originated as the Innovative Medicines Initiative (IMI), but it is now called the Innovative Health Initiative (IHI) [23]. Projects approved by the IMI/IHI allow for bundling efforts and knowledge in different environments, making the collection and evaluation of information much more efficient and less costly for each entity involved in the research. 

RSV infection has been included in the initiatives of two IMI projects: Rescue [24] and Promise [25]. It is therefore expected to see some fruitful outcomes from that partnership. This is certainly the case for Rescue, whose main focus was to investigate the burden of disease among infants and children. The Promise project—a continuation of the Rescue program—may have a greater focus on the elderly, but unfortunately most of the data will only be available/published in 2024. Although collaboration is the objective of Promise, it has been challenging to procure common outputs in time, mainly caused by the COVID-19 pandemic, which significantly delayed the study. 

The point to make here is that vaccine companies want to launch onto the market They do not want to wait for the full RSV package of information of Promise to become available. Meanwhile, those IMI projects present very interesting approaches to tackle complex health(care) problems, and to enhance their strength, the availability of their results could be made conditional for the local reimbursement of new products. But it is noticed that those programs are not free of challenges once approved (e.g., finding the right compromises between operating differences and work cultures of industry and academia). IMI projects often start with very ambitious goals that end up not being reachable with the assigned budget, which needs to be adjusted over time. Management of the whole project can be a very delicate entrepreneurship, especially if the program encompasses much differed attentions such as randomized clinical trials, modeling evaluations, and systematic literature reviews [26]. 

Because of scheduling issues with Promise IMI and the pressure of presenting the data sooner rather than later, sponsoring companies are thrown into that difficult situation estimating the burden of RSV burden. Their focus is on what they consider to be critical about the disease and their vaccine, which often is a partial view. They have now indirectly created a big “puzzle” to be solved when estimating the true RSV burden, based on the small blocks of disintegrated information they disclosed. Looking at the overall objective, which is to estimate the total burden into a clear context-related environment of respiratory infectious diseases in ageing adults, there must be more efficient ways to reach that endpoint. This would likely include better planning and a more rigorous approach towards following the guidelines that are available for conducting these studies. 

In summary, studies on the burden of disease for any illness should be planned and conducted well in advance through a unified approach and collaboration among the different stakeholders (public and private). This is crucial when little variation is expected in the results by whomever is conducting the assessment. This is expected to bring greater efficiency to the process as well as more reliable end results. For RSV specifically, this approach should have been obvious, especially since so many vaccine producers started operating at approximately the same time (i.e., more than 5 years prior to the launch of the vaccine). 

This is the lesson learned for moving forward about a preferred pathway on the investigation of the burden of illness studies for any future infection, such as clostridium, staphylococcus, gonorrhea, among others. The healthcare industry requires a clear compass to guide researchers into uncharted waters to avoid being adrift on a sea of confusing information.

## Figures and Tables

**Figure 1:**
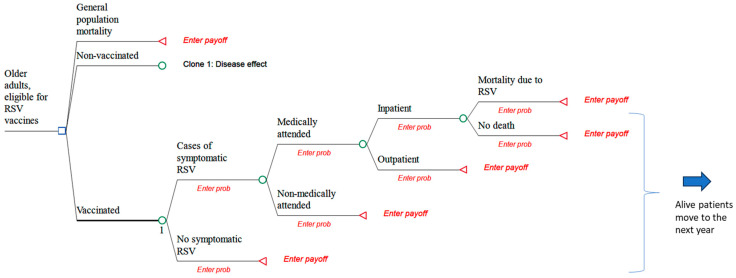
Replica of the model structure presented in the paper [10].
